# Self-Controlled Video Feedback Facilitates the Learning of Tactical Skills in Tennis

**DOI:** 10.1080/02701367.2023.2275801

**Published:** 2023-12-15

**Authors:** Bart R. van der Meer, Michel A. C. van den Hoven, John van der Kamp, Geert J. P. Savelsbergh

**Affiliations:** a Vrije Universiteit Amsterdam; b ROC Nova College; c Windesheim University of Applied Sciences; d Amsterdam Institute of Sport Science

**Keywords:** Motor learning, self-control, self-controlled feedback, self-regulation, tactical skills, tennis, video feedback

## Abstract

**Purpose:** This study aimed to examine the effect of self-controlled video feedback on the learning of tactical motor skills in tennis, and additionally, whether this was affected by learners’ self-efficacy and self-regulative skills. **Method:** Twenty-three intermediately skilled tennis players were assigned to either a self-controlled group that was provided video feedback on request or a yoked group that received an identical, externally controlled video feedback schedule. In three training sessions participants practiced serve and volley play. Video feedback with attentional cueing and transitional statements that focused solely on individual tactical gameplay was provided by a licensed tennis coach. Individual tactical performance was measured with a custom designed Tactical Tennis Tool (TTT) in a pretest, posttest and in a one-week retention test. Before each test self-efficacy was measured, and a questionnaire was administered to measure self-regulative skills. **Results:** Analyses revealed significantly larger improvements in tactical performance relative to the pretest for the self-controlled group than for the yoked group in both the posttest and the one-week retention test. No differences were found in self-efficacy. Finally, the improvements in tactical performance were not predicted by self-efficacy and/or self-regulative skills. **Conclusion:** The advantage of self-controlled video feedback extends to the learning of a complex tactical task in tennis. Future research should verify the observed benefits of a self-controlled learning environment in comparison to a coach-controlled learning environment.

Providing feedback effectively is highly relevant for supporting the acquisition of sport skills (Huet et al., 2009). One crucial aspect to increase performance is the feedback schedule, that it is the timing and frequency of feedback provision (Chiviacowsky & Wulf, 2007). In recent years, researchers have demonstrated that giving learners control of when to receive feedback increases learning compared to externally controlled conditions, where a coach (or experimenter) decides and provides feedback. For instance, self-controlled feedback has been shown to improve performance in complex skills such as executing a sequence of techniques in taekwondo (Goudini et al., 2019), performing a jump-landing task (Benjaminse et al., 2017), shooting in basketball (Aiken et al., 2012), chipping in golf (Post et al., 2016), jumping the mini-trampoline (Ste-Marie et al., 2013), weightlifting (Souissi et al., 2021), overhand service in volleyball (Zetou et al., 2018) and swimming (Marques & Corrêa, 2016). Self-controlled feedback has been shown beneficial for both children (e.g., Janelle et al., 1997; Ste-Marie et al., 2013; Zetou et al., 2018) and adults (e.g., Aiken et al., 2012; Goudini et al., 2019). Furthermore, self-controlled feedback provided as knowledge of performance (KP) (e.g., Aiken et al., 2012; Goudini et al., 2019; Ste-Marie et al., 2013) and as knowledge of results (KR) (e.g., Chiviacowsky et al., 2008; Januário et al., 2019) have both been shown to benefit learning. Overall, there is increasing evidence that self-controlled feedback benefits learning, although the strength of this evidence has been questioned by a recent meta-analysis (McKay et al., 2022), but for a meta-analysis with a different conclusion, see (Jimenez-Diaz et al., 2021). The effects of self-controlled feedback are typically examined by comparing learning in a group of learners that are free to choose when to receive feedback to a yoked group in which learners receive feedback on the same schedules as their matched participant in the self-controlled group. Because timing and frequency of feedback are similar between groups, differences in learning can be attributed to the opportunity to decide whether or not to obtain feedback during practice.

Despite an increasing amount of evidence showing benefits of self-controlled feedback when learning *technical* motor skills, only one study investigated the effect of self-controlled feedback on acquiring *tactical* skills. van Maarseveen et al. (2018) investigated the effect of self-controlled group video feedback on learning tactical skills in small-sided soccer games. Highly talented female youth soccer players received video feedback about their offensive performance in 3 versus 2 attacks. Results indicated that players in the self-controlled group were more actively involved in the discussions directly following the video feedback. However, no significant learning benefits from self-control compared to the externally controlled yoked group were found in the one-week retention test. Maarseveen et al.’s study differs from most other research on self-controlled feedback in two ways. First, feedback was requested by a group, whereas typically feedback is requested and provided individually. Secondly, solely highly skilled participants were included such that the potential for noticeable improvements in performance following a small number of practice sessions were relatively small. Hence, in our study we examined intermediate players who received video feedback about their tactical performance individually, the primary aim being to examine the effect of self-controlled feedback on the learning of tactical motor skills.

Several studies investigating self-controlled learning took Zimmerman’s self-regulation of learning model (Zimmerman, 2000) as the theoretical framework to understand the potential benefits of self-controlled feedback (e.g., Janelle et al., 1997; Kok et al., 2020; Ste-Marie et al., 2013, 2016). Zimmerman’s model regards self-regulative learning as going through multiple cycles of three separable though interdependent phases, that is, the forethought phase, the performance phase and the self-reflection phase. In the forethought phase learners analyze the task, set their goals and plan how to reach those goals strategically. Several motivational beliefs, such as self-efficacy and intrinsic interest strengthen this process because it leads to setting more difficult goals and more persistent striving to achieve these goals. In the performance phase learners execute the task during which they use self-control and self-observation to monitor their performance. Self-controlled (video) feedback manifests itself mostly in this phase by supporting self-observation. Providing learners with autonomy to choose when to obtain feedback does provide guidance for the learning, but in an environment that encourages to gather this guidance actively. Finally, in the self-reflection phase learners self-judge their task-execution and attribute positive and negative causes to their performance. This causes self-reactions that consequently influence learners’ forethought phase in the next learning cycle.

One influential explanation for the benefits of self-regulation on learning can be derived from self-determination theory (Deci & Ryan, 2000). It holds that higher perceived autonomy, which can stem from self-controlled feedback, would increase motivational beliefs (such as self-efficacy and task interest; for a similar account see OPTIMAL by Wulf & Lewthwaite, 2016). Accordingly, several studies (Grand et al., 2015; Ste-Marie et al., 2013, 2016) investigated the relationship between self-efficacy and performance following self-controlled feedback. For instance, Ste-Marie et al. (2013) investigated children learning a progression of trampoline skills. Children in the self-controlled group chose when to self-observe their practice performance via video. After practice, they outperformed children in the yoked group for whom video feedback via self-observation was imposed. A hierarchical multiple regression analysis was used to show that self-control as well as self-efficacy (at retention) significantly predicted performance at retention (see also Grand et al., 2015; Ste-Marie et al., 2013, 2016). However, Zimmerman’s self-regulation model (2000) emphasizes that it is the motivational beliefs *during* practice that should increase motor learning (i.e., the improvements in performance from pretest to retention). Instead, a positive relationship between performance in retention and self-efficacy might merely reflect the typically observed momentarily associations between motivational beliefs and performance (Rosenqvist & Skans, 2015; Ste-Marie et al., 2013; Wulf et al., 2012). Therefore, more evidence is needed to explain the purported positive effects of self-control on the motivational beliefs *during* practice and subsequently on learning. To the best of our knowledge only one study (Kok et al., 2020) examined this relationship between motivational beliefs during practice and learning (i.e., as reflected in the relatively permanent improvements in performance from pretest to retention). They compared a self-controlled video feedback group of secondary school students, a yoked video feedback group and a “traditional” teacher guided feedback group practicing a shot put across four PE-lessons. Self-efficacy was measured before the pretest and retention but also during practice, before each lesson. Kok et al. (2020) found that self-efficacy during practice was indeed a significant predictor of learning the shot-put technique. Yet, there were no learning differences between groups. In the present study, we further assessed the contribution of self-efficacy during practice for learning (i.e., improvements in performance from pretest to retention).

Finally, self-regulative skills such as planning, self-monitoring, evaluation and reflection can be learned (e.g., see Schunk & Zimmerman, 1998) and thus improved over time. It is likely that individuals differ in the degree to which they master and apply these skills, and thus in the degree to which their learning profits from self-regulation (Kok et al., 2020; Zimmerman, 2000), and thus from learning environments that are designed to promote self-regulation, such as for example self-controlled feedback. This possible relationship, however, has not been examined directly. Hence, in the present study, we used a validated questionnaire that measures the relatively stable self-regulative skills of an individual (Toering et al., 2012) to examine whether the more general self-regulative skills of planning, self-monitoring, evaluation, reflection, motivation and self-efficacy predict learning of tactical motor skills.

To sum up, the main aim of this study was to investigate whether self-controlled video feedback enhanced learning of tactical motor skills in tennis. Intermediately skilled tennis players were quasi-randomly divided in a self-controlled feedback group (SC) and a yoked group (YK). In three training sessions participants practiced serve and volley play. A licensed tennis coach provided video feedback with attentional cueing and transitional statements that focused solely on individual tactical gameplay. Tactical performance was measured in a pretest, posttest (i.e., during the final practice session) and a one-week retention test. Self-efficacy was measured during the tests and the practice sessions and self-regulative skills were measured once after retention. We hypothesized that players in the self-controlled group would show greater tactical learning (i.e., improvement in tactical performance from pretest to retention) compared to players in the yoked group. Furthermore, we hypothesized that self-control, self-efficacy during practice and self-regulative skills would predict players learning (i.e., from pretest to retention), but not or to lesser extent, players’ more immediate performance improvements during practice (i.e., from pretest to posttest), mainly because immediate performance improvement during practice not always predict learning (i.e., performance improvements from pretest to retention, Salmoni et al., 1984; Schmidt & Bjork, 1992).

## Method

### Participants

A priori power-analysis (G*Power 3.1.9) revealed 24 participants would be needed to measure a moderate effect using an analysis of variance comparing two groups in tow repeated tests (i.e., α = .05, β = .8, effect size f = 0.3). Twenty-eight adult participants (12 female 16 male) were recruited to participate during several tennis trainings in the region of Den Bosch, The Netherlands. Five participants did not attend all practice sessions due to injuries (*n* = 2) and Covid-related symptoms (*n* = 3) and were excluded from analysis. The remaining 23 participants (11 female, 12 male) had an average age of 43.3 years (SD = 16.2) and were intermediate tennis players with an average of 13.5 (SD = 5.9) years of experience in playing tennis and 8.9 (SD = 5.7) years of playing tennis competition. Participants’ tennis doubles (2 versus 2) performance levels (Median = 7.4, SD = 1, range 5.34–8.99) were based on the Royal Dutch Lawn Tennis Association (“KNLTB”) system ranging from 1 (performing at international level) to 9 (performing at the lowest recreational level). Written informed consent was obtained prior to the pretest. The experiment was approved by the Scientific and Ethical Review Board of the Faculty of Behavioural and Movement Sciences, VU Amsterdam.

### Materials

To accurately represent normal tennis conditions, practice sessions were performed on a typical outside gravel tennis court with dimensions prescribed by the International Tennis Federation (ITF). Tests and practice sessions were recorded by fixing a GoPro camera (Hero 5, GoPro Inc., San Mateo, California, USA) to a fence 4 m behind the baseline at a height of 2.5 m. This elevated video position was used in order to provide a clear overview and to aid in perceiving depth (Mann et al., 2009). Video recordings were used to analyze tactical performance. The camera was Wi-Fi connected to an iPad (10 inch (2017), 32 GB, Apple, Cupertino, California, USA) to enable instant video feedback.

Individual performance scores for serve and volley play were quantified using an especially designed Tactical Tennis Tool (i.e., TTT). Starting point for developing the TTT were the parameters that describe relevant behaviors in serve and volley as used in match analyses by the Royal Dutch Lawn Tennis Association (KNLTB). In addition, we used an observation tool by McPherson and French (1991, see also Nielsen & McPherson, 2001) that consists of “decision rules for coding components of tennis performance during the serve and game play following the serve” (p. 548). The TTT describes 2 (first or second serve) × 3 (ball placement) × 4 (intention) × 6 (recovery position) = 144 possible tactical behaviors for serve, and 6 (court position) × 9 (ball placement) × 4 (intention) × 9 (recovery position) = 1944 possible tactical behaviors for rally play. These were all scored from 1 to 10 based on tactical performance ratings by a coach with more than 15 years of experience at international level and by an embedded scientist of the KNLTB. To bolster the use of TTT, different types of reliability and validity were determined. A research assistant coded 80 trials three times. This showed that intra-observer reliability was high (average measure ICC = .996, F (19) = 249.98, *p* < .05). Also, two certified tennis coaches and the first author (who is an amateur tennis player) independently scored the same 80 trials showing that inter-observer reliability was high (i.e., average measure ICC = .800, F (77) = 4.99, *p* < .05). Content validity was good. The correlation between TTT scores and the scores of the McPherson tool, (ρ = ranging from .316 to .595, *p* < .05) showed fair to moderate concurrent validity. This can be attributed to the McPherson tool incorporating technical execution whereas the TTT solely measures tactical performance. For a more detailed description see Supplementary materials.

Following Bandura’s (2006) guidelines, self-efficacy was measured prior to each test (see also Ste-Marie et al., 2013). Participants were asked to rate two statements between 1 (completely disagree/bad) and 10 (totally agree/good), specifically “I can play serve and volley” and “I feel confident playing serve and volley”. The average score of these two questions was taken as the participants’ perceived self-efficacy.

A validated Dutch questionnaire (Toering et al., 2012) was used to measure self-regulative skills. The questionnaire provides six separate scores for subscales that are considered important in Zimmerman’s (2000) model of self-regulation (i.e., planning, self-monitoring, evaluation, reflection, effort and self-efficacy). The average score of these subscales constituted a total score for self-regulative skills. The questionnaire was filled in by all participants after the retention test on a laptop (HP ProBook 430 G6, 13.3 inch).

### Design and procedure

Participants were quasi-randomly assigned to a self-controlled group (SC) or a yoked group (YK) by first ranking them from high to low on tennis doubles performance based on their KNLTB performance levels, and then alternately assigning them to the SC group and to the YK group. Finally, it was verified whether genders were divided equally between groups.

Participants followed a three-week training program. Each week participants performed one practice session of 20 trials that lasted approximately 10–15 minutes and was preceded by a short warm up. In each practice trial participants were expected to play serve and volley to try and win the point. Also, each participant fulfilled the role of doubles partner during another participant’s 20 trials. These dyads were similar in all practice sessions and were formed based on availability in time, hence the dyads were not always from the same experimental group. Two experienced tennis players played as opponents. They were instructed to adapt their performance to the level of the participant. Standard tennis doubles rules applied. Each trial was recorded to enable direct video feedback. Furthermore, the winner of the point was noted by the researcher which enabled distinguishing between successful (i.e., point won) and unsuccessful (i.e., point lost) trials.

To improve serve and volley play, participants were individually (i.e., without the doubles partner) provided with video feedback augmented with the coaches’ instructions solely about the tactical aspects of their serve and volley performance by an experienced and certified tennis coach. Specifically, the coach, based on his expert judgment, provided verbal cueing and transitional statements about stroke direction, intention of the stroke, positioning after the stroke and running lines of their last trial. Transitional statements were, for instance, “try to run more forward after you serve so that the return does not bounce and you keep pressure on the opponents” or “after serving through the center, run a little more to the outside in order to successfully return strokes to the outside”. After each trial, participants in the SC group were allowed to choose whether they wanted to receive video feedback with the coach’s verbal cueing and transitional statements. The participants in the YK group received video feedback in a yoked schedule, seemingly on the initiative of the coach but provided such that the scheduling of feedback was equal between groups. Accordingly, feedback for the YK group was always provided after the corresponding trial of the participant they were yoked with. For instance, if the participant in the SC group asked for feedback after trial 2, 7, 9 and 15 the yoked player also received video feedback with the coach’s verbal cueing and transitional statements after his or her 2^nd^, 7^th^, 9^th^ and 15^th^ trial respectively. This required the participants in the SC group to always be involved in an earlier practice session then their yoked counterparts. Other from this, practice conditions of the YK group were exactly the same as for the SC group, that is, the same court, same practice duration, same tennis coach, and similar verbal cues and transitional statements.

**Figure 1.**
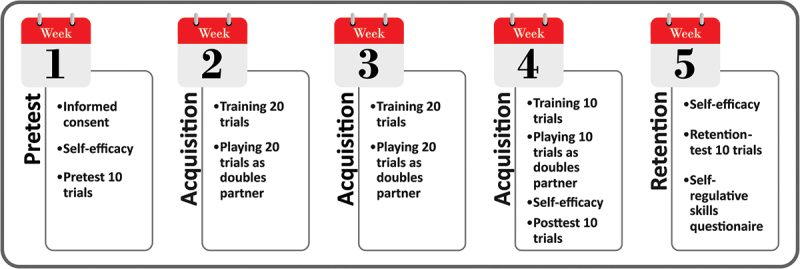
Study design.

A total of three tests, consisting of ten trials each, were conducted. Specifically, a pretest one week prior to the first practice session, a posttest directly after the third practice session and a retention test one week thereafter (Figure 1). The retention test was performed to measure the relatively permanent change in tactical skills (Schmidt & Lee, 2005). In the third practice session just 10 trials were performed to avoid the influence of fatigue in the posttest. In all tests, three experienced tennis players complemented the participant to play doubles. Similar to practice, these complementing players were instructed to adapt their performance to the level of the participant. During the tests no feedback was provided. Video recordings were made to measure performance using the TTT afterward. Prior to all tests self-efficacy was measured. After the retention test, a questionnaire was administered to measure self-regulative skills.

### Statistical analyses

All statistical analyses were performed with IBM statistics SPSS, version 27. Significance level was set at .05. The variables were tested to assess normality (Shapiro—Wilk Test), homoscedasticity (Levene’s test) and sphericity (Mauchly’s test) (*p* > .05). Additionally, for the regression analyses variables were also tested for multicollinearity, linearity and independence.

First, a repeated measures ANOVA was used to examine the number of feedback request during practice across block of five trials for the SC group only. Next, perusal of the data suggested considerable interindividual differences in pretest TTT-score that were apparently associated with group. To control for these interindividual differences, we deemed conducting an ANCOVA more appropriate than the originally planned ANOVA with repeated measures, even though an independent t-test showed the difference did not reach significance (t(21) = −1.6, *p* = .12). Hence, to compare the immediate performance improvements during practice between the two groups, the difference in TTT-score from pretest to posttest was submitted to an analysis of covariance with group (SC versus YK) as fixed factor and pretest score as the covariate. Similarly, and more critically, to compare learning, or the relatively permanent improvement in performance between groups, the difference in TTT-score from pretest to retention test was submitted to an analysis of covariance with group (SC versus YK) as fixed factor and pretest score as the covariate. Finally, self-efficacy scores were submitted to a repeated measures ANOVA with group (SC and YK) as a between-subject factor and test as a within factor. Effect sizes were reported as partial eta squared (η^2^) and were interpreted as small for η^2^ < .06, medium for η^2^ < .14 and large for η^2^ > .14 (Cohen, 1969).

Finally, a hierarchical linear regression was conducted to predict immediate performance improvements during practice (i.e., difference in TTT-score between pretest and posttest) based on group, self-efficacy during practice and self-regulative skills. Group (SC versus YK) was entered in the first step to define a basic model, while in the second step self-efficacy during practice and self-regulative skills were entered. A similar hierarchical regression was performed with learning (i.e., difference in TTT-score between pretest and retention) as outcome variable.

## Results

### Feedback

**Figure 2.**
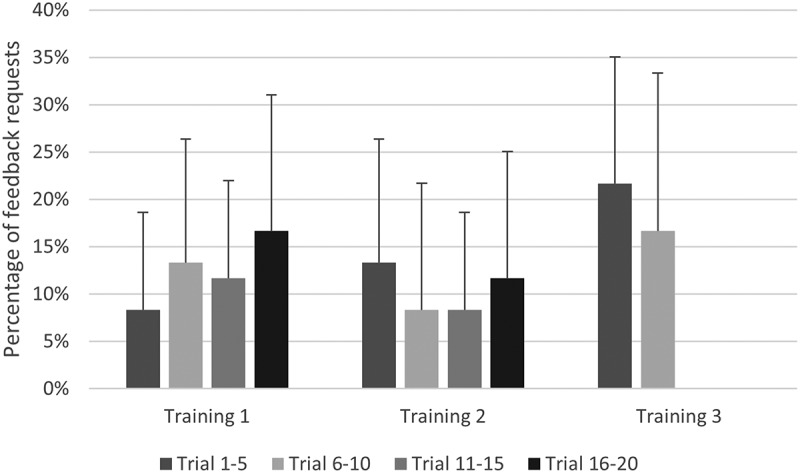
Percentage of feedback requests per block of 5 trials (error bars represent standard deviations).

The SC group requested feedback in 12.7% of all trials (i.e., approximately once in every 8 trials). A RM-ANOVA did not show significant differences in the number of feedback requests across blocks of 5 practice trials, F (5.71, 62.82) = 1.43, *p* > .05, η_p_^2^ = 0.115 (Figure 2).

Participants in the SC group won 29.9% of all points and 70.1% were lost. Feedback was requested mostly after a point was lost (i.e., 94.7%) and only 5.3% of feedback was requested after the participant won the point.

### Tactical score

**Figure 3.**
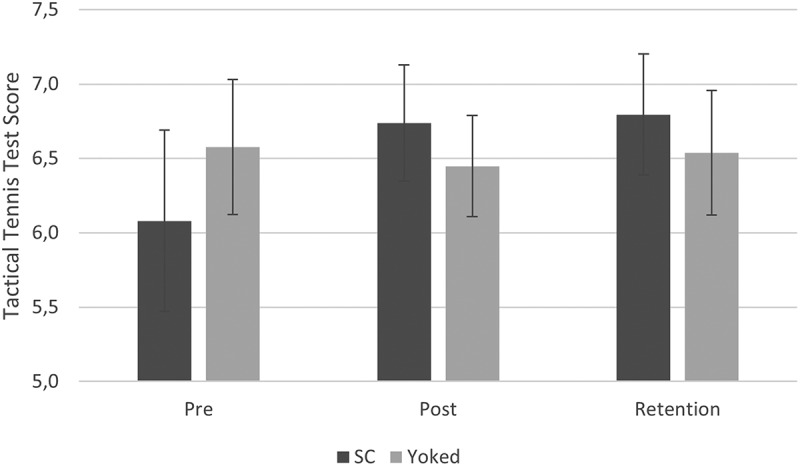
TTT score in pretest, posttest and retention test for the SC group and YK group (error bars represent standard deviations).

Figure 3 suggests an increase in TTT from pretest to posttest (i.e., immediate performance improvements during practice) and from pretest to retention (i.e., learning) for the SC group but not for the YK group. This was confirmed by an ANCOVA with the pretest score as covariate and the difference in TTT score between pretest and posttest as dependent variable. That is, a significant effect of the covariate was found, F (1, 20) = 12.1, *p* < .05, η_p_^2^ = .38, indicating that the difference in TTT score between pretest and posttest was smaller with higher pretest scores. Most importantly, however, there was a significant effect of group, F (1, 20) = 4.36, *p* < .05, η_p_^2^ = .18 indicating larger immediate performance improvements during practice for the SC group than for the YK group. The second ANCOVA showed similar results for the difference in TTT score between the pretest and retention test. That is, a significant effect of the covariate was found, F (1, 20) = 10.7, *p* < .05, η_p_^2^ = .35, indicating that learning was smaller with higher pretest scores. Again, there was a significant effect of group, F (1, 20) = 4.6, *p* < .05, η_p_^2^ = .19 indicating increased learning for the SC group than the YK group.

### Self-efficacy

**Figure 4.**
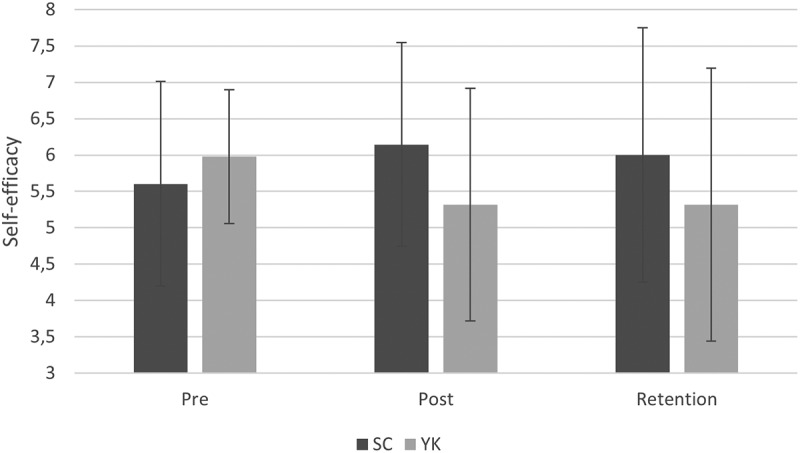
Self-efficacy in pretest, posttest and retention test for SC group and YK group (error bars represent standard deviations).

Figure 4 suggests an increase in reported self-efficacy from pretest to posttest and from pretest to retention in the SC group, while self-efficacy appears to decrease in the YK group. Except for the SC group in the posttest, all variables were normally distributed and therefore a repeated measures ANOVA was performed. This showed that there were no significant main effects for group, F (1, 21) = 0.44, *p* > .05, η_p_^2^ = .02 and test, F (2, 42) = 0.09, *p* > .05, η_p_^2^ = .04. In addition, also the interaction between test and group was not significant, F (2, 42) = 2.1, *p* = .14, η_p_^2^ = .09.

### Self-regulative skills

Self-regulative skills were not normally distributed. A Spearman correlation showed no significant correlation in the SC group between self-regulative skills and the difference in TTT score between pretest and posttest (i.e., immediate improvements in practice performance), r (10) = .007, *p* > .05. Additionally, immediate improvements in practice performance was not significantly related to any subscale of self-regulative skills (i.e. planning, r (10) = −.077, *p* > .05; monitoring, r (10) = −.461, *p* > .05; evaluation, r (10) = .227, *p* > .05; reflection, r (10) = −.316, *p* > .05; motivation, r (10) = −.049, *p* > .05; general self-efficacy, r (10) = .466, *p* > .05). Similarly, a Spearman correlation showed no significant correlation in the SC group between self-regulative skills and difference in TTT score between pretest and retention (i.e., learning), r (10) = .007, *p* > .05. Additionally, learning was not significantly related to any subscale of self-regulative skills (i.e., planning, r (10) = −.081, *p* > .05; monitoring, r (10) = .098, *p* > .05; evaluation, r (10) = .113, *p* > .05; reflection, r (10) = −.135, *p* > .05; motivation, r (10) = −.221, *p* > .05; general self-efficacy, r (10) = .141, *p* > .05).

### Predicting immediate improvement in practice performance and learning

A multiple linear regression was performed to predict the difference in TTT score between pretest and posttest (i.e., improvement in practice performance) based on group, self-efficacy during practice and self-regulative skills. A significant regression equation was found after step 1, F (1,21) = 6.93, *p* < .05 with an adjusted R^2^ of .212 (Table 1). Inclusion of the variables self-efficacy during practice and self-regulative skills in step 2 did not lead to a significant improvement in the prediction model, ∆ adjusted R^2^ = .038, *p* > .05.

**Table 1.** Multiple regression for difference in TTT score between pretest and posttest (i.e., immediate improvement in practice performance).

	B	β	[95% CI]	p	Adjusted R^2^	∆ Adjusted R^2^
Step 1					.21 (*p* = .004)	
Constant	.658					
Group	−.787	−.498*	[−1.408, −0.165]	.016*		
Step 2					.25 (*p* = .266)	.038 (*p* = .242)
Constant	−2.042					
Group	−.791	−.501*	[−1.424, −0.159]	.017*		
Self-efficacy during practice	−.041	−.167	[−0.145, 0.063]	.418		
Self-regulative skills	.156	.337	[−.034, 0.345]	.102		

A second hierarchical linear regression was calculated to predict the difference in TTT score between pretest and retention (i.e., learning) based on group, self-efficacy during practice and self-regulative skills. A significant regression equation was found, F (1,21) = 8.03 *p* < .05 with an adjusted R^2^ of .242 (Table 2). Inclusion of the variables self-efficacy during practice and self-regulative skills in step 2 did not lead to a significant improvement in the prediction model, ∆ adjusted R^2^ = .005, *p* > .05.

**Table 2.** Multiple regression for difference in TTT score between pretest and retention (i.e., learning).

	B	β	[95% CI]	p	Adjusted R^2^	∆ Adjusted R^2^
Step 1					.242 (*p* = .001)	
Constant	0.713					
Group	−0.766	−0.526*	[−1.327, −0.204]	.010*		
Step 2					.247 (*p* = .657)	.005 (*p* = .363)
Constant	−.743					
Group	−.811	−.557*	[−1.395, −0.227]	.009*		
Self-efficacy during practice	−.053	−.231	[−0.149, 0.044]	.267		
Self-regulative skills	.102	.239	[−.073, 0.277]	.237		

## Discussion

Previous studies showed that self-controlled video feedback promotes motor skill learning. However, the learning of *tactical* skills in sports has not received a great deal of attention. The aim of this study was therefore to examine whether self-controlled video feedback improved the learning of tactical skills in tennis. It was found that the self-controlled group showed significantly larger practice improvements and learned significantly more than its yoked counterpart, indicating that self-controlled video feedback also provides an advantage when learning complex tactical skills in tennis. Contrary to expectations, self-efficacy during practice and self-regulative skills did not predict players learning, and also not their immediate performance improvements during practice.

This study is the first to show that allowing participants to self-control timing and frequency of video feedback is advantageous for learning tactical skills. This is consistent with previous studies in technical motor skills (for reviews, see Sanli et al., 2013; Wulf, 2007) but was not shown earlier for tactical skills (van Maarseveen et al., 2018). The current study showed that compared to the external control of feedback provision, self-control increased both improvement in practice performance and resulted in relatively permanent learning. That is, the immediate practice advantage of the SC group sustained after one-week retention. This is an important first empirical finding, but these positive effects on tactical skill learning need to be further examined to increase its practical value. Typically, in sports, it is the coach who decides when and what feedback to provide, based on their content and didactical knowledge combined with the observations of the athlete’s actions. Hence, subsequent research should also compare learning in a self-controlled group with a coach-controlled feedback group, because in all likelihood, this would lead to a more fitting feedback schedule than the mere yoking. Indeed, Kok et al. (2020) showed that in acquisition of the shot put, self-controlled feedback did lead to similar but not superior learning advantages as teacher-controlled feedback among secondary school students. Further to this point, the current study did neither include a control group, which did not practice at all, nor placebo group, which received a similar but task irrelevant intervention. Without these groups, the conclusions necessarily remain tentative. Yet, the current observations suggest it well worth investing the (large) effort to conduct a full-blown design.

The present study did not resolve what constraints underpin the observed advantages of self-controlled feedback. In line with Zimmerman’s model of self-regulation, self-efficacy has been suggested as an important candidate in this respect. Indeed, a fair amount of studies suggested that self-efficacy is associated with learning (Janelle et al., 1997; Kok et al., 2020; Rosenqvist & Skans, 2015; Ste-Marie et al., 2013; Wulf et al., 2012), but typically these studies correlated self-efficacy after practice to performance after practice. However, based on Zimmerman’s (2000) self-regulation model (Kok et al., 2020; Ryan & Deci, 2000), it is self-efficacy during practice or acquisition that should be the main impetus for learning. Yet, self-efficacy during practice was not found to predict learning (and also not the immediate performance improvements during practice). Therefore, more research is needed in which also other motivational beliefs are measured such as autonomy (e.g. see self-determination theory Ryan & Deci, 2000; OPTIMAL theory Wulf & Lewthwaite, 2016), task interest and intrinsic motivation (Grand et al., 2015; Ste-Marie et al., 2013), perceived success (Ste-Marie et al., 2013) or sense of agency (Haggard & Tsakiris, 2009). Finally, we were also unable to find evidence that participants’ general self-regulation skills related to learning. However, it cannot be ruled out that this lack is related to the nature of the questionnaire, which focuses more on relatively stable attributes or dispositions of general skills than on task specific experiences or perception (Toering et al., 2012). In this respect, it is notable that one of the subscales of the questionnaire that measures (general) self-efficacy did not show a relationship with our task specific measure of self-efficacy.^1^^1^Spearman correlation between general self-efficacy and task-specific self-efficacy score in the retention test was not significant, r (22) = .094, *p* > .05.

Our results showed that both during training and between trainings there was no decrease in the amount of requested feedback (“no fading schedule”). This is in sharp contrast with other research, since most other studies that report the number of feedback requests across practice do, in fact, observe a fading schedule (Aiken et al., 2012; Jalalvand et al., 2019; Post et al., 2016; Ste-Marie et al., 2013; Wulf et al., 2005). The current study distinguishes itself from other studies into self-controlled feedback by the high complexity of the tactical task. And even though the participants had relatively high amounts of tennis experience, we, arguably, hypothesize that this complexity (relative to skill level) is likely the main reason that no fading schedule was observed. This idea is supported by the observation that the self-efficacy was similar before pretest and after retention, suggesting that participants did not feel much more competent after training, and no decrease in the desire for feedback occurred, even though the three training sessions presented a longer training period than in most comparable research. In this respect, it may also be relevant to consider the type of feedback. Possibly, the type of feedback (i.e., KR versus KP) influences whether learners prefer to ask feedback after more or less successful practice trials. In the current study KP was almost exclusively requested after less successful trials (i.e., in 94.7% this was after a point was lost).^2^^2^Note that the high amount of feedback requests after unsuccessful trials in our study (94.7%) should be interpreted cautiously. Requesting feedback after a point was lost or won may not be equivalent to the learner perceiving the trial to be successful or unsuccessful. Similarly, Nunes et al. (2019) and Post et al. (2016) noted more feedback requests after unsuccessful trials than after successful trials. Yet, since Janelle’s first study (1997) on self-controlled feedback, the dominant view is that participants tend to ask feedback more frequently after successful trials (e.g., Benjaminse et al., 2017; Chiviacowsky et al., 2008; Fairbrother et al., 2012; Figueiredo et al., 2018; Wulf & Lewthwaite, 2016). This is typically explained by a supposed concomitant increase in perceived competence or self-efficacy (Chiviacowsky, 2014; Chiviacowsky et al., 2012; Figueiredo et al., 2018). However, this effect may be restricted to studies that provided KR. Studies that provided KP (e.g., Nunes et al., 2019; Post et al., 2016), including the present study, seem to report more feedback requests after unsuccessful trials. They also seem to involve the more complex learning tasks. It may be that receiving positive feedback is less important for evidently complex learning tasks (e.g., van Maarseveen et al., 2018), that is, participants may more easily accept that they performed suboptimal because the task and the feedback is complex, and hence, are also less likely to reduce requests for feedback during practice. Future research should try to uncover whether the task complexity and/or type of feedback effects the timing of feedback request in self-controlled learning environments.

In conclusion, this study is the first to demonstrate that the advantage of self-controlled video feedback extends to the learning of a complex tactical task in tennis. More research is needed to examine what individual constraints other than self-efficacy and self-regulative skills underpin this advantage.

## Supplementary Material

Reliability and Validity of the Tactical Tennis Tool (TTT)

## References

[cit0001] Aiken, C. A., Fairbrother, J. T., & Post, P. G. (2012). The effects of self-controlled video feedback on the learning of the basketball set shot. *Frontiers in Psychology*, 3(338), 1–8. 10.3389/fpsyg.2012.00338 22973257 PMC3438820

[cit0002] Bandura, A. (2006). Chapter 14: Guide for contrstucting self-efficacy scales. In F. Pajares & T. Urdan (Eds.), *Self-efficacy beliefs of adolescents* (pp. 307–337). Information Age Publishing.

[cit0003] Benjaminse, A., Janssen, I., Postma, W., & Otten, B. (2017). 2D video feedback improves landing technique in elite female handball players. *British Journal of Sports Medicine*, 51(4), 294.3–295. 10.1136/bjsports-2016-097372.28

[cit0004] Chiviacowsky, S. (2014). Self-controlled practice: Autonomy protects perceptions of competence and enhances motor learning. *Psychology of Sport and Exercise*, 15(5), 505–510. 10.1016/j.psychsport.2014.05.003

[cit0005] Chiviacowsky, S., & Wulf, G. (2007). Feedback after good trials enhances learning. *Research Quarterly for Exercise and Sport*, 78(1), 40–47. 10.1080/02701367.2007.10599402 17479573

[cit0006] Chiviacowsky, S., Wulf, G., de Medeiros, F. L., Kaefer, A., & Tani, G. (2008). Learning benefits of self-controlled knowledge of results in 10-year-old children. *Research Quarterly for Exercise and Sport*, 79(3), 405–410. 10.1080/02701367.2008.10599505 18816953

[cit0007] Chiviacowsky, S., Wulf, G., & Lewthwaite, R. (2012). Self-controlled learning: The importance of protecting perceptions of competence. *Frontiers in Psychology*, 3(458), 1–8. 10.3389/fpsyg.2012.00458 23130006 PMC3487418

[cit0008] Cohen, J. (1969). *Statistical power analysis for the behavioural sciences*. Academic Press.

[cit0009] Deci, E. L., & Ryan, R. M. (2000). The “what” and “why” of goal pursuits: Human needs and the self-determination of behavior. *Psychological Inquiry*, 11(4), 227–268. 10.1207/S15327965PLI1104_01

[cit0010] Fairbrother, J. T., Laughlin, D. D., & Nguyen, T. V. (2012). Self-controlled feedback facilitates motor learning in both high and low activity individuals. *Frontiers in Psychology*, 3, 1–8. 10.3389/fpsyg.2012.00323 22969745 PMC3431613

[cit0011] Figueiredo, L. S., Ugrinowitsch, H., Freire, A. B., Shea, J. B., & Benda, R. N. (2018). External control of knowledge of results: Learner involvement enhances motor skill transfer. *Perceptual and Motor Skills*, 125(2), 400–416. 10.1177/0031512517753503 29350078

[cit0012] Goudini, R., Ashrafpoornavaee, S., & Farsi, A. (2019). The effects of self-controlled and instructor-controlled feedback on motor learning and intrinsic motivation among novice adolescent taekwondo players. *Acta Gymnica*, 49(1), 33–39. 10.5507/AG.2019.002

[cit0013] Grand, K. F., Bruzi, A. T., Dyke, F. B., Godwin, M. M., Leiker, A. M., Thompson, A. G., Buchanan, T. L., & Miller, M. W. (2015). Why self-controlled feedback enhances motor learning: Answers from electroencephalography and indices of motivation. *Human Movement Science*, 43, 23–32. 10.1016/j.humov.2015.06.013 26163375

[cit0014] Haggard, P., & Tsakiris, M. (2009). The experience of agency. *Current Directions in Psychological Science*, 18(4), 242–246. 10.1111/j.1467-8721.2009.01644.x

[cit0015] Huet, M., Camachon, C., Fernandez, L., Jacobs, D. M., & Montagne, G. (2009). Self-controlled concurrent feedback and the education of attention towards perceptual invariants. *Human Movement Science*, 28(4), 450–467. 10.1016/j.humov.2008.12.004 19394099

[cit0016] Jalalvand, M., Bahram, A., Daneshfar, A., & Arsham, S. (2019). The effect of gradual self-control of task difficulty and feedback on learning golf putting. *Research Quarterly for Exercise and Sport*, 90(4), 429–439. 10.1080/02701367.2019.1612510 31329023

[cit0017] Janelle, C. M., Barba, D. A., Frehlich, S. G., Tennant, L. K., & Cauraugh, J. H. (1997). Maximizing performance feedback effectiveness through videotape replay and a self-controlled learning environment. *Research Quarterly for Exercise and Sport*, 68(4), 269–279. 10.1080/02701367.1997.10608008 9421839

[cit0018] Januário, M. S., Figueiredo, L. S., Portes, L. L., & Benda, R. N. (2019). Effects of self-controlled knowledge of results on learning a taekwondo serial skill. *Perceptual and Motor Skills*, 126(6), 1178–1194. 10.1177/0031512519869086 31422740

[cit0019] Jimenez-Diaz, J., Chaves-Castro, K., & Morera-Castro, M. (2021). Effect of self-controlled and regulated feedback on motor skill performance and learning: A meta-analytic study. *Journal of Motor Behavior*, 53(3), 385–398. 10.1080/00222895.2020.1782825 32623973

[cit0020] Kok, M., Komen, A., van Capelleveen, L., & van der Kamp, J. (2020). The effects of self-controlled video feedback on motor learning and self-efficacy in a physical education setting: An exploratory study on the shot-put. *Physical Education and Sport Pedagogy*, 25(1), 49–66. 10.1080/17408989.2019.1688773

[cit0021] Mann, D. L., Farrow, D., Shuttleworth, R., & Hopwood, M. (2009). The influence of viewing perspective on decision-making and visual search behaviour in an invasive sport. *International Journal of Sport Psychology*, 40, 546–564.

[cit0022] Marques, P. G., & Corrêa, U. C. (2016). The effect of learner’s control of self-observation strategies on learning of front crawl. *Acta Psychologica*, 164, 151–156. 10.1016/j.actpsy.2016.01.006 26821171

[cit0023] McKay, B., Yantha, Z. D., Hussien, J., Carter, M. J., & Ste-Marie, D. M. (2022). Meta-analytic findings of the self-controlled motor learning literature: Underpowered, biased, and lacking evidential value. *Meta-Psychology*, 6. https://orcid.org/0000-0003-1851-7609

[cit0024] McPherson, S. L., & French, K. E. (1991). Changes in cognitive strategies and motor skill in tennis. *Journal of Sport & Exercise Psychology*, 13(1), 26–41. 10.1123/jsep.13.1.26

[cit0025] Nielsen, T. M., & McPherson, S. L. (2001). Response selection and execution skills of professionals and novices during singles tennis competition. *Perceptual and Motor Skills*, 93(2), 541–555. 10.2466/pms.2001.93.2.541 11769911

[cit0026] Nunes, M. E., Correa, U. C., Souza, M. G. T. X., Basso, L., Coelho, D., & Santos, S. (2019). No improvement on the learning of golf putting by older persons with self-controlled knowledge of performance. *Journal of Aging and Phyiscal Activity*, 27(3), 300–308. 10.1123/japa.2018-0053 30160582

[cit0027] Post, P. G., Aiken, C. A., Laughlin, D. D., & Fairbrother, J. T. (2016). Self-control over combined video feedback and modeling facilitates motor learning. *Human Movement Science*, 47, 49–59. 10.1016/j.humov.2016.01.014 26874750

[cit0028] Rosenqvist, O., & Skans, O. N. (2015). Confidence enhanced performance? - The causal effects of success on future performance in professional golf tournaments. *Journal of Economic Behavior and Organization*, 117, 281–295. 10.1016/j.jebo.2015.06.020

[cit0029] Ryan, R. M., & Deci, E. L. (2000). Self-determination theory and the facilitation of intrinsic motivation, social development, and well-being. *American Psychological Association*, 55(1), 68–78. 10.1037/0003-066X.55.1.68 11392867

[cit0030] Salmoni, A. W., Schmidt, R. A., & Walter, C. B. (1984). Knowledge of results and motor learning: A review and critical reappraisal. *Swine Health and Productions*, 95(3), 355–386. 10.1037/0033-2909.95.3.355 6399752

[cit0031] Sanli, E. A., Patterson, J. T., Bray, S. R., & Lee, T. D. (2013). Understanding self-controlled motor learning protocols through the self-determination theory. *Frontiers in Psychology*, 3(611), 1–17. 10.3389/fpsyg.2012.00611 PMC3576889 23430980

[cit0032] Schmidt, R. A., & Bjork, R. A. (1992). New conceptualizations of practice: Common principles in three paradigms suggest new concepts for training. *Psychological Science*, 3(4), 207–217. 10.1111/j.1467-9280.1992.tb00029.x

[cit0033] Schmidt, R. A., & Lee, T. D. (2005). *Motor control and learning: A behavioral emphasis* (4th ed.). Human Kinetics.

[cit0034] Schunk, D. H., & Zimmerman, B. J. (1998). *Self-regulated learning: From teaching to self-reflective practice*. Guilford Press.

[cit0035] Souissi, M. A., Souissi, H., Elghoul, Y., Masmoudi, L., Trabelsi, O., Ammar, A., Chtourou, H., & Souissi, N. (2021). Information processing and technical knowledge contribute to self-controlled video feedback for children learning the snatch movement in weightlifting. *Perceptual and Motor Skills*, 128(4), 1785–1805. 10.1177/00315125211011728 33910395

[cit0036] Ste-Marie, D. M., Carter, M. J., Law, B., Vertes, K., & Smith, V. (2016). Self-controlled learning benefits: Exploring contributions of self-efficacy and intrinsic motivation via path analysis. *Journal of Sports Sciences*, 34(17), 1650–1656. 10.1080/02640414.2015.1130236 26707002

[cit0037] Ste-Marie, D. M., Vertes, K. A., Law, B., & Rymal, A. M. (2013). Learner-controlled self-observation is advantageous for motor skill acquisition. *Frontiers in Psychology*, 3(556), 1–10. 10.3389/fpsyg.2012.00556 PMC3554505 23355826

[cit0038] Toering, T., Elferink-Gemser, M. T., Jonker, L., van Heuvelen, M. J. G., & Visscher, C. (2012). Measuring self-regulation in a learning context: Reliability and validity of the Self-Regulation of Learning Self-Report Scale (SRL-SRS). *International Journal of Sport and Exercise Psychology*, 10(1), 24–38. 10.1080/1612197X.2012.645132

[cit0039] van Maarseveen, M. J. J., Oudejans, R. R. D., & Savelsbergh, G. J. P. (2018). Self-controlled video feedback on tactical skills for soccer teams results in more active involvement of players. *Human Movement Science*, 57, 194–204. 10.1016/j.humov.2017.12.005 29253741

[cit0040] Wulf, G. (2007). Self-controlled practice enhances motor learning: Implications for physiotherapy. *Physiotherapy*, 93(2), 96–101. 10.1016/j.physio.2006.08.005

[cit0041] Wulf, G., Chiviacowsky, S., & Lewthwaite, R. (2012). Altering mindset can enhance motor learning in older adults. *Psychology and Aging*, 27(1), 14–21. 10.1037/a0025718 21988153

[cit0042] Wulf, G., & Lewthwaite, R. (2016). Optimizing performance through intrinsic motivation and attention for learning: The OPTIMAL theory of motor learning. *Psychonomic Bulletin and Review*, 23(5), 1382–1414. 10.3758/s13423-015-0999-9 26833314

[cit0043] Wulf, G., Raupach, M., & Pfeiffer, F. (2005). Self-controlled observational practice enhances learning. *Research Quarterly for Exercise and Sport*, 76(1), 107–111. 10.1080/02701367.2005.10599266 15810775

[cit0044] Zetou, E., Vernadakis, N., Mountaki, F., & Karypidou, D. (2018). The effect of self-regulated feedback on acquisition and learning the overhand service skill of novice female athletes in volleyball. *Cuadernos de Psicología Del Deporte*, 18(1), 221–228.

[cit0045] Zimmerman, B. J. (2000). Chapter 2: Attening self-regulation. A social cognitive perspective. In M. Boekaerts, P. R. Pintrich, & M. Zeidner (Eds.), *Handbook of self-regulation* (pp. 13–39). Academic Press. 10.1016/B978-012109890-2/50031-7

