# Supplementary material for: Self-Controlled Video Feedback Facilitates the Learning of Tactical Skills in Tennis
Source: Res Q Exerc Sport. 2023 Dec 15;95(2):537–45. doi: 10.1080/02701367.2023.2275801 (PMC11147453; doi:10.1080/02701367.2023.2275801)
Supplement: Reliability and Validity of the Tactical Tennis Tool (TTT) [file URQE_A_2275801_SM0214.docx]

**Supplementary materials: Reliability and validity of the Tactical Tennis Tool (TTT)**

**Introduction**

In tennis, observation tools exist that measure technical *and* tactical performance (e.g. McPherson & French, 1991). However, none is available that uniquely measures tactical performance. Hence, to measure tactical performance, we developed the Tactical Tennis Tool (TTT). The TTT is an encompassing instrument that evaluates on court tactical decision making in serve and volley play. Here, we report the details of the TTT, including inter- and intra-rater reliability and content, concurrent and construct validity.

**Method & Results**

***Notation system***

Starting point for developing the TTT were the parameters of serve and volley behaviours in the match analyses by the Royal Dutch Lawn Tennis Association (‘KNLTB’). This was complemented with the observation tool by McPherson & French (1991, see also Nielsen & McPherson, 2001), which consists of “decision rules for coding components of tennis performance during the serve and game play following the serve” (p. 548)

The TTT distinguishes 2 (first or second serve) x 3 (ball placement) x 4 (intention) x 6 (recovery position) = 144 possible tactical behaviours for the serve, plus 6 (court position) x 9 (ball placement) x 4 (intention) x 9 (recovery position) = 1944 possible tactical behaviours for rally play. These more than 2000 behaviours were all assigned a score on an ordinal 10-point scale, indicating tactical performance level of each of behaviour. These scores were assigned separately by an embedded scientist of the KNLTB and a coach with more than 15 years of experience at international level. In case they did not agree about the assigned score, they discussed until consensus was reached. Figure A1 shows an example of all possible TTT-scores, when the player places his 1^st^ serve in the T-zone of the service area.^[[1]](#footnote-1)^

Using the TTT requires analysis of video footage. Every stroke of the tennis player is analysed by denoting the corresponding combination of events, thus identifying a unique tactical behaviour (Figure A2) corresponding to a set score (see above). The overall score for tactical decision making of one played point is calculated by averaging the score of all strokes within the point or rally (N.B., the strokes of the opponent(s) and doubles partner are not included).


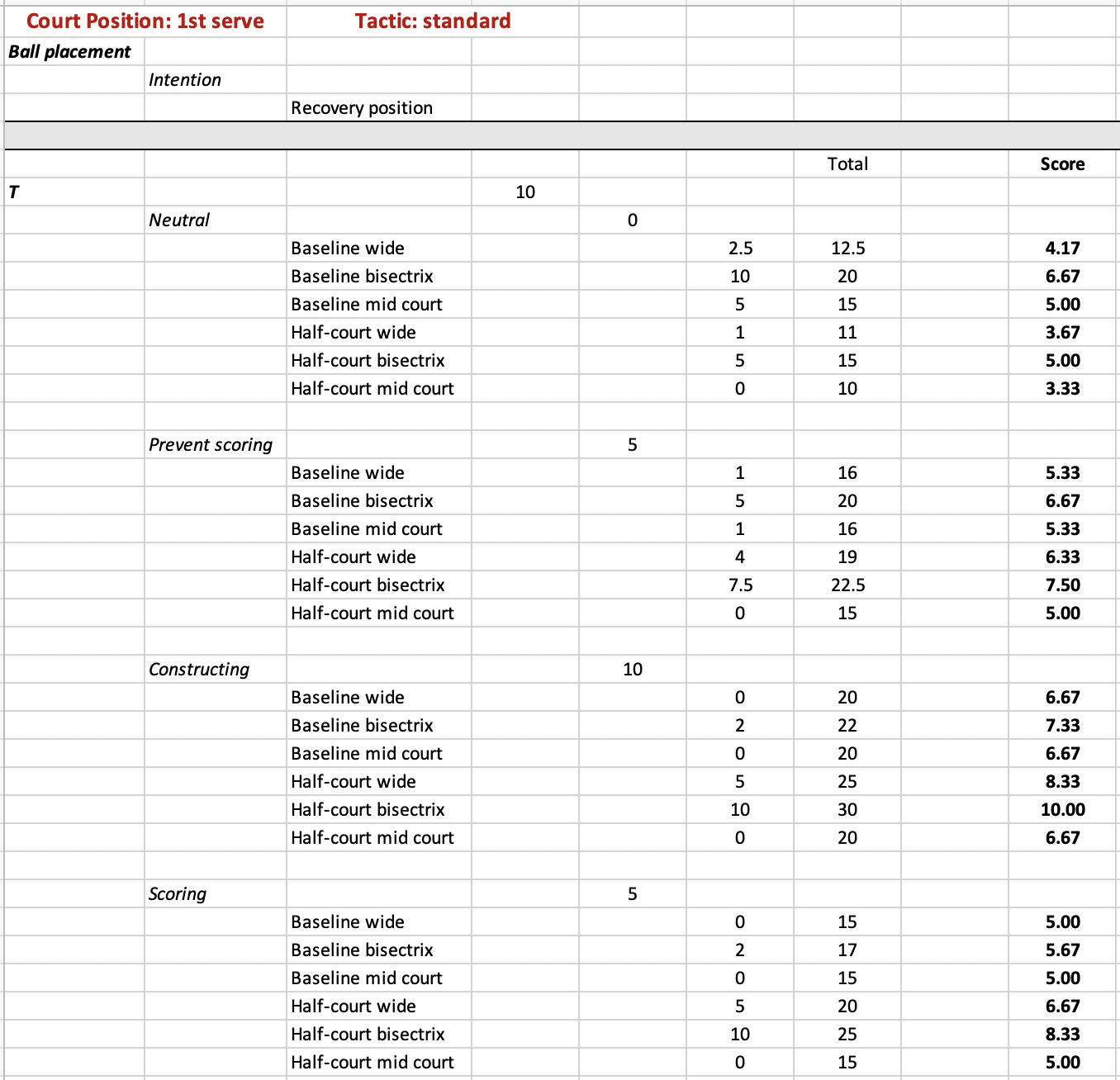
Figure A1: Example of scores for all possible tactical behaviours and corresponding scores when a first serve is placed in the T-zone of the service area.

Point number

Code manually entered after assessing the video

Score per stroke

Average score for this point

| **1** |  |
| --- | --- |
| B235 | 7.50 |
| 4428 | 8.00 |
| 8918 | 5.33 |
|  |  |
|  | **6.94** |

Ball placement

Recovery position

b235

Serve / court position

Intention

*Figure A2: Explanation of coding in the TTT*

***Participants***

Serve and volley performances of tennis doubles players on recreational and professional level were assessed, using recorded video footage of eight players (recreational *n* = 4, professional *n* = 4). The recreational players (2 male, 2 female) were instructed to play serve and volley during practice sessions and for the professional players (4 male) video footage of official matches was used. Recreational participants’ skill level was Universal Tennis Rating < 9.0 and professional participants’ skill level was ATP ranking > 50. Recreational participants had an average age of 32 years (SD = 0.8), and professional players were on average 31 years (SD = 5.0) of age.

***Stimulus materials***

To assess intra- and inter-rater reliability, and content, concurrent and construct validity, a total of 160 serve and volley points were analysed (i.e., 20 per player). All matches were recorded from approximately 5 m behind the baseline and at a minimum height of 2 m. One rater is a researcher, one is a certified tennis coach (KNLTB KSS 3) and one is both a researcher and a certified tennis coach (KNLTB KSS 3). All scoring was performed individually.

*Reliability*

All ICC estimates and their 95% confidence intervals were calculated using SPSS statistical package version 27 based on 2-way random-effects model, single rater and absolute-agreement. Values less than .5 are indicative of poor reliability, values between .5 and .75 indicate moderate reliability, values between .75 and .9 indicate good reliability, and values greater than .9 indicate excellent reliability (Koo & Li, 2016).

*Intra-rater reliability.* A total of 80 trials (all recreational trials) were coded three times by one of the researchers to determine intra-rater reliability. The average measure ICC was .996 with a 95% confidence interval from .992 to .998, F(19) = 249.977, p < .05, indicating an excellent intra-rater reliability (Koo & Li, 2016).

*Interrater reliability.* A total of 20 trials were coded by three raters to assess interrater reliability. The average measure ICC was .800 with a 95% confidence interval from .719 to .862, F(77) = 4.988, p < .05, indicating a good reliability between the raters (Koo & Li, 2016). An excellent reliability (average measure ICC = .976, 95% confidence interval from .940 to .991, F(19) = 42.225, p < .05) was calculated between the performance measurements of the two tennis coaches, indicating that the TTT is more reliable and thus preferably coded by an assessor with tennis experience.

*Content validity*.

Content validity was secured by developing the TTT together with the embedded scientist of the Dutch Royal Lawn Tennis Association (‘KNLTB’) and a certified KNLTB KSS 5 coach (i.e., equivalent to ITF level 3 coach), who has approximately 20 years of experience in coaching tennis at national and international level. They assigned scores (and if necessary discussed disagreements) for the tactical behaviours (see above) for all possible situations that the TTT distinguishes. They both approved the final version of the TTT.

*Concurrent validity.* Concurrent validity was determined by correlating the results of the TTT to the best available reference assessment, that is, the McPherson tool (McPherson & French, 1991). One researcher scored the TTT tool and the McPherson tool for the same 80 recreational trials. A Shapiro-Wilk test showed a significant departure from normality for both datasets, hence Spearman’s Rho (*ρ*) correlations were calculated between the scores of TTT and of the McPherson tool. *ρ* ranged from .316 to .595, *p* < .05 indicating a fair to moderate correlation between the TTT sores and the scores of the McPherson tool. Most likely, this suboptimal correlation is best explained by the fact that the McPherson tool takes also technical skill into account whereas the TTT solely measures tactical skill.

*Construct validity.* TTT scores of recreational and professional players were compared to determine if the notation system was able to differentiate between skill level. An independent samples t-test showed a significant difference in the scores for recreational (*M* = 7.08, *SD* = 0.19) and professional (*M* = 8.92, *SD* = 0.10) skill levels, *t*(38) = 8.61, *p* < .05, indicating that the TTT is able to differentiate between recreational and professional players.

**Discussion and conclusion**

The TTT showed excellent intra-rater reliability and good inter-rater reliability, good content validity, moderate concurrent validity and good construct validity. The TTT is therefore a valuable tool for measuring tactical skills in serve and volley play in tennis, both in practice sessions and in match situations.

***References***

Koo, T. K., & Li, M. Y. (2016). A Guideline of Selecting and Reporting Intraclass Correlation Coefficients for Reliability Research. *Journal of Chiropractic Medicine*, *15*(2), 155–163. https://doi.org/10.1016/j.jcm.2016.02.012

McPherson, S. L., & French, K. E. (1991). Changes in Cognitive Strategies and Motor Skill in Tennis. *Journal of Sport & Exercise Psychology*, *13*, 26–41.

Nielsen, T. M., & McPherson, S. L. (2001). Response selection and execution skills of professionals and novices during singles tennis competition. *Perceptual and Motor Skills*, *93*(2), 541–555. https://doi.org/10.2466/pms.2001.93.2.541

1. Those interested in all scores can contact the first author at [bmeer@novacollege.nl](mailto:bmeer@novacollege.nl) [↑](#footnote-ref-1)
